# Sensitive Skin in the Indian Population: An Epidemiological Approach

**DOI:** 10.3389/fmed.2019.00029

**Published:** 2019-02-20

**Authors:** Emilie Brenaut, Laurent Misery, Charles Taieb

**Affiliations:** ^1^Department of Dermatology, University Hospital of Brest, Brest, France; ^2^Université de Bretagne Occidentale, LIEN, Brest, France; ^3^EMMA, Direction Scientifique, Fontenay sous Bois, France; ^4^Hôpital Necker Enfants Malades, Santé Publique, Paris, France

**Keywords:** sensitive skin, reactive skin, India, epidemiological study, phone survey, skin disorder, environmental factors, cosmetics

## Abstract

Sensitive skin is a very frequent condition, the prevalence of this syndrome has been studied in different countries in Europe, in United States and in Japan. The aim of the study was to evaluate the epidemiology of sensitive skin in the Indian population, like this has never been studied in this country. A representative nationwide sample of the Indian population aged 15 and over was selected. Individuals were selected as per the quota method (based on sex, age, householder profession, rural/urban location, and region). In total, 27.9% of men and 36.7% of women declared having “sensitive” or “very sensitive” skin. The difference between the 2 sexes was very significant. Of these, 5.1% of men and 7.2% of women reported having “very sensitive” skin. The subjects complaining about “sensitive” or “very sensitive” skin were 2–4 times more likely to declare suffering from atopic dermatitis, acne, psoriasis, or vitiligo. They were 2 to 3 times more reactive to climatic factors, environmental factors, cosmetics and food intake. In conclusion, although less frequently reported than in other countries, sensitive skin is a frequent condition in India, affecting about one third of the population.

## Introduction

The concept of sensitive skin was introduced by Frosch (1) and Thiers (2). Sensitive skin is characterized by the occurrence of sensations of tingling, prickling, heat, burning, pain, or itching, and occasionally by erythema in response to multiple physical (UV radiation, heat, cold, or wind), chemical (cosmetics, soaps, pollution, or water), psychological (stress), and/or hormonal (menstrual cycle) factors that should not provoke such sensations (3–6). Sensitive skin is also called reactive, hyper-reactive, intolerant or irritable skin. The term “sensitive skin” mainly refers to facial skin, but this condition can also affect other areas of the body, such as the hands, scalp, or genital area (6–8).

The first epidemiological study of sensitive skin was conducted in the United Kingdom in 2001 (7); then, studies were conducted in many countries throughout the world, including Belgium, China, France, Germany, Greece, Italy, Portugal, Spain, Switzerland, the United States of America, Brazil, Japan, and Russia (8–13). These studies used similar methodologies based on surveys of samples of the population aged 15 years and older. It is very important to obtain data on sensitive skin in different countries and there was no data in India.

The aim of the present study was to perform the first epidemiological study on sensitive skin in India.

## Patients and methods

As in previous studies (8–13), questionnaires were administered by CSA Santé (CSA Health Institute) and the same methodology was used. Data were collected between April and May.

A sample of 3,012 individuals was selected based on criteria that allow representation of the Indian population aged 15 and older. The participants were selected by the quota method (based on gender, age, occupation of the family head, agglomeration level, and region). Systematic monitoring of interviews was conducted with a repeat call to 20% of those interviewed. In case this process could lead to a different outcome of any type, even for only one survey, all interviews conducted by the same interviewer were double-checked by another individual. No error was detected. Interviews were conducted in English or local languages: Hindi, Marathi, Gujarati, Bengali, or Tamil.

The first part of the questionnaire was related to demographics (geographical area, age, gender, and social status) and to skin type. The second part addressed facial skin sensitivity. The subjects were requested to rate their skin as “very sensitive,” “sensitive,” “slightly sensitive,” or “not sensitive.” The interviewees responded to an open question regarding their perception of the onset of tingling, burning or irritation in the presence of different factors, such as emotional stimuli, cold, heat, sun exposure, cosmetics, dry air, air-conditioning, water, air pollution, and variations in temperature. The participants were also asked if they had ever suffered from rashes without an apparent cause, if their facial skin was easily irritated, if they had visited a dermatologist during the previous year, and whether they had any skin diseases.

To characterize sensitive skin in the Indian population, the characteristics of the subjects with “sensitive” or “very sensitive” skin (the “sensitive skin” group) were compared with those of the subjects having “not very sensitive” or “not sensitive at all” skin (“non-sensitive skin” group).

Quantitative variables were compared between groups with Student's *t*-test (when there were two groups to compare) or ANOVA (when there were more than two groups to compare). When the conditions for the application of these tests were not met, the Wilcoxon and Kruskal-Wallis non-parametric tests were performed. Categorical variables were compared with an appropriate test, with Fisher's test used if the conditions were not completely met. Statistical analyses were performed with SAS software version 8.2 (SAS Institute).

Ethical review and approval were not required for this study in accordance with the local legislation and institutional requirements.

## Results

### Sensitive Skin and Demographic Data

**Figure 1:**
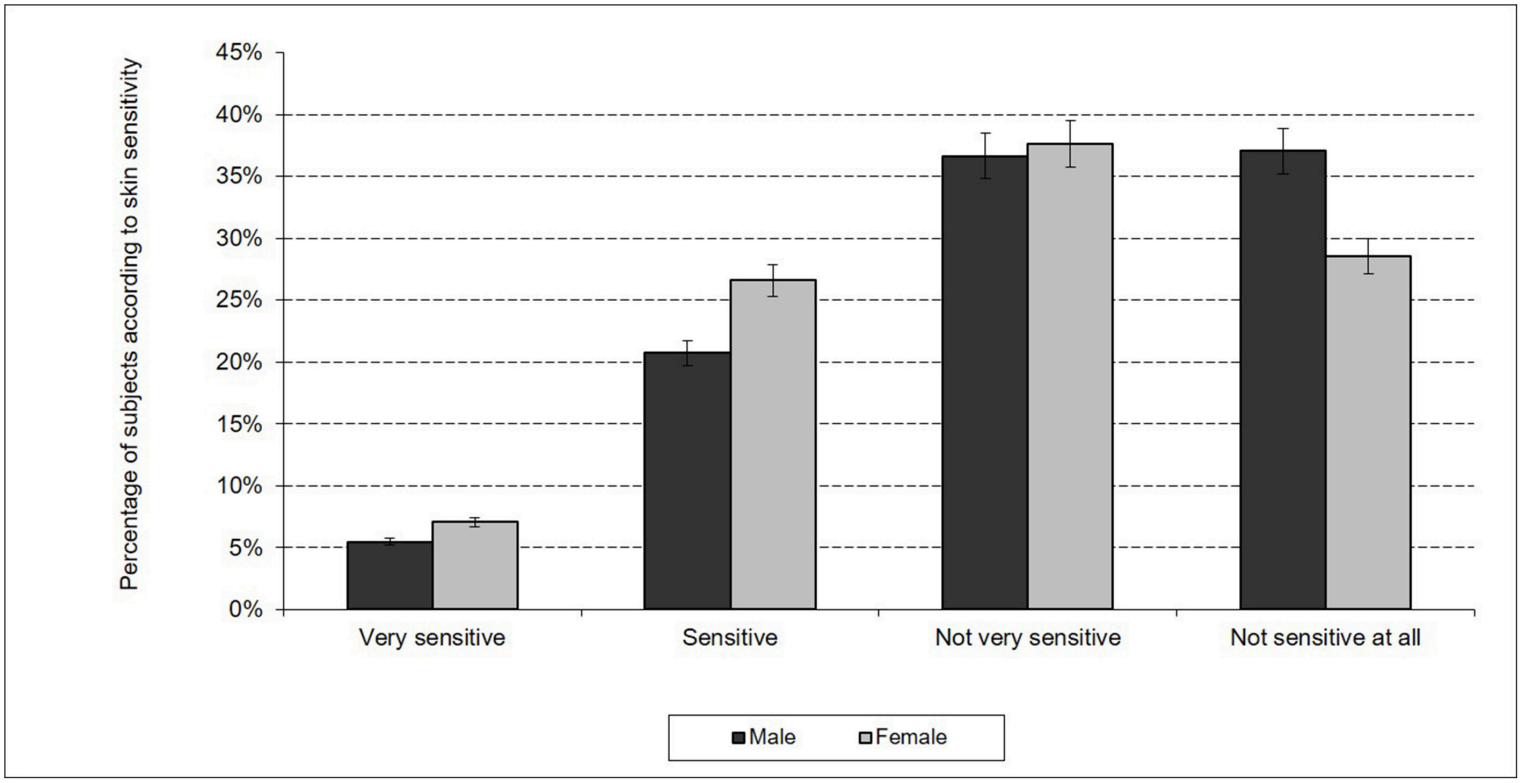
Skin sensitivity in India according sex.

In total, 27.9% of men and 36.7% of women reported having “sensitive” or “very sensitive” skin (Figure 1). The difference between the 2 sexes was significant (Student's *t*-test, *p* < 0.001). Of these, 5.5% of men and 8.2% of women reported having “very sensitive skin.”

**Figure 2:**
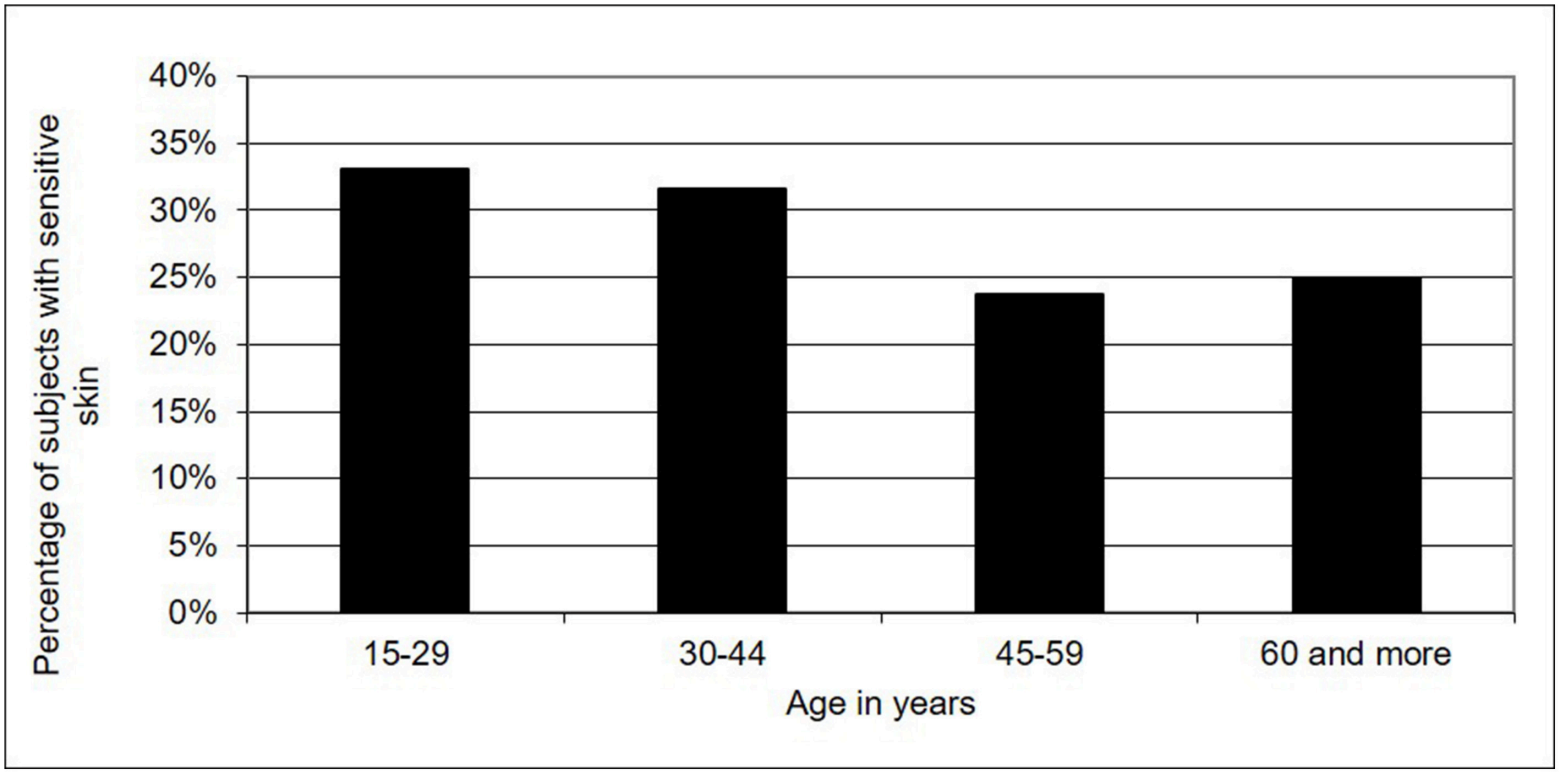
Skin sensitivity in India according age.

We detected significant differences in skin sensitivity according to age (Figure 2). Indeed, younger subjects (between 15 and 44 years old) more frequently reported sensitive skin than older subjects (over 45 years old) did (32.4 vs. 24.2%, *p* < 0.001). The overall non-response rate was approximately 0.2% for men and women. This rate was higher in older subjects (0.9% in subjects over 60 years old vs. 0.0% in subjects between 30 and 59 years old vs. 0.1% in subjects under 29 years old). The non-response rate suggested that the term “sensitive skin” had meaning for the majority of the sample of the Indian population.

### Sensitive Skin and Skin Disorders

**Figure 3:**
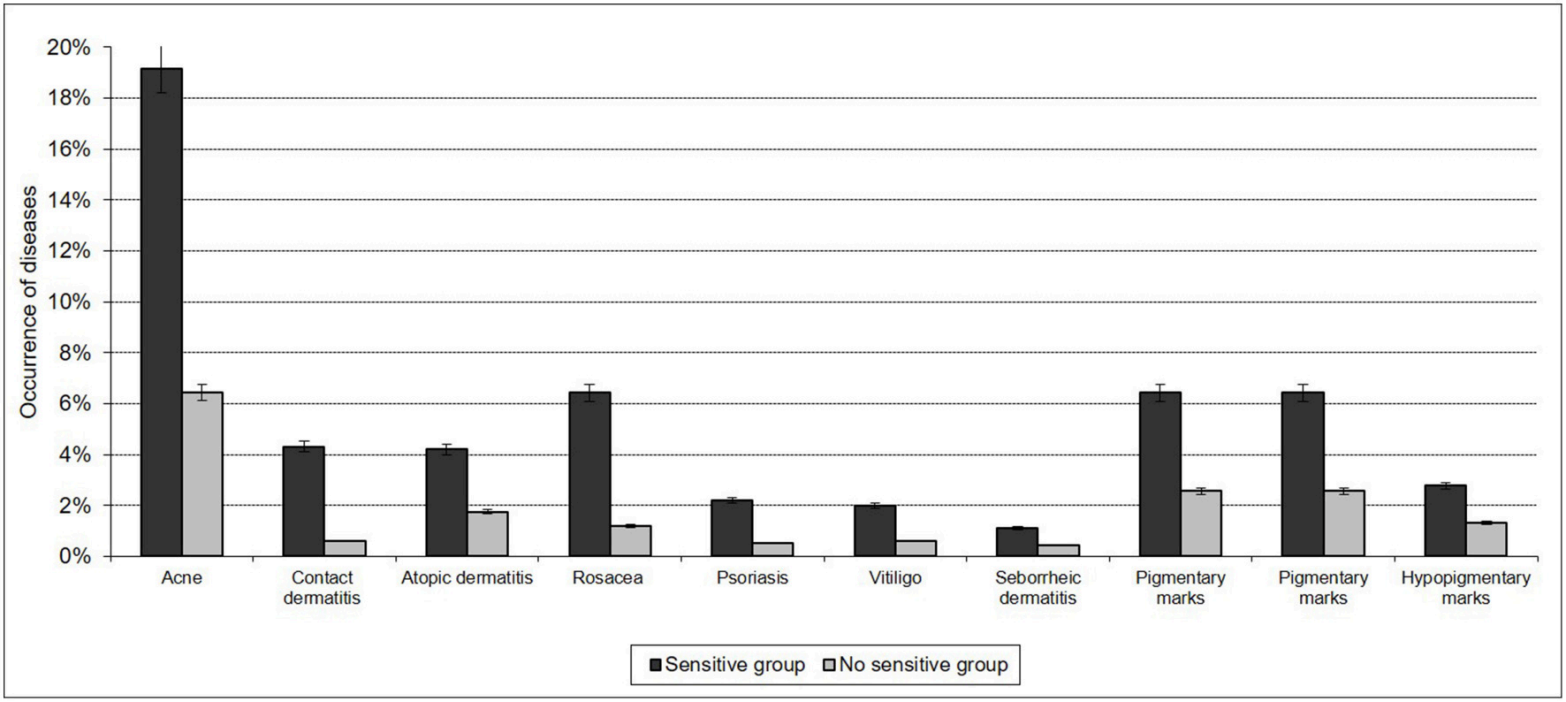
Sensitive skin and reported skin disorders.

An evaluation of the frequency of various dermatoses showed differences between the 2 groups. The subjects in the “sensitive skin” group were 3 times more likely to declare suffering from a dermatosis (44.9 vs. 15.0%). Indeed, the subjects complaining about “sensitive” skin were 2–4 times more likely to report atopic dermatitis, acne, psoriasis, vitiligo, rosacea, or contact dermatitis compared with the subjects in the “non-sensitive skin” group (Figure 3).

A history of atopic dermatitis or eczema in the childhood was more frequent in the subjects in the “sensitive skin” group (11.8 vs. 6.3%, *p* < 0.001).

Interviewees who declared that they had dry, oily, or combination skin were also significantly more numerous (*p* < 0.0001) in the “sensitive skin” group than those with normal skin were.

In total, 42% of people with dry skin, 39% of people with oily skin, and 28% of people with combination skin described “sensitive” or “very sensitive” skin. The prevalence of “sensitive” or “very sensitive” skin among the subjects with normal skin was 22%.

### Skin Sensitivity According to Climatic and Environmental Factors

**Figure 4:**
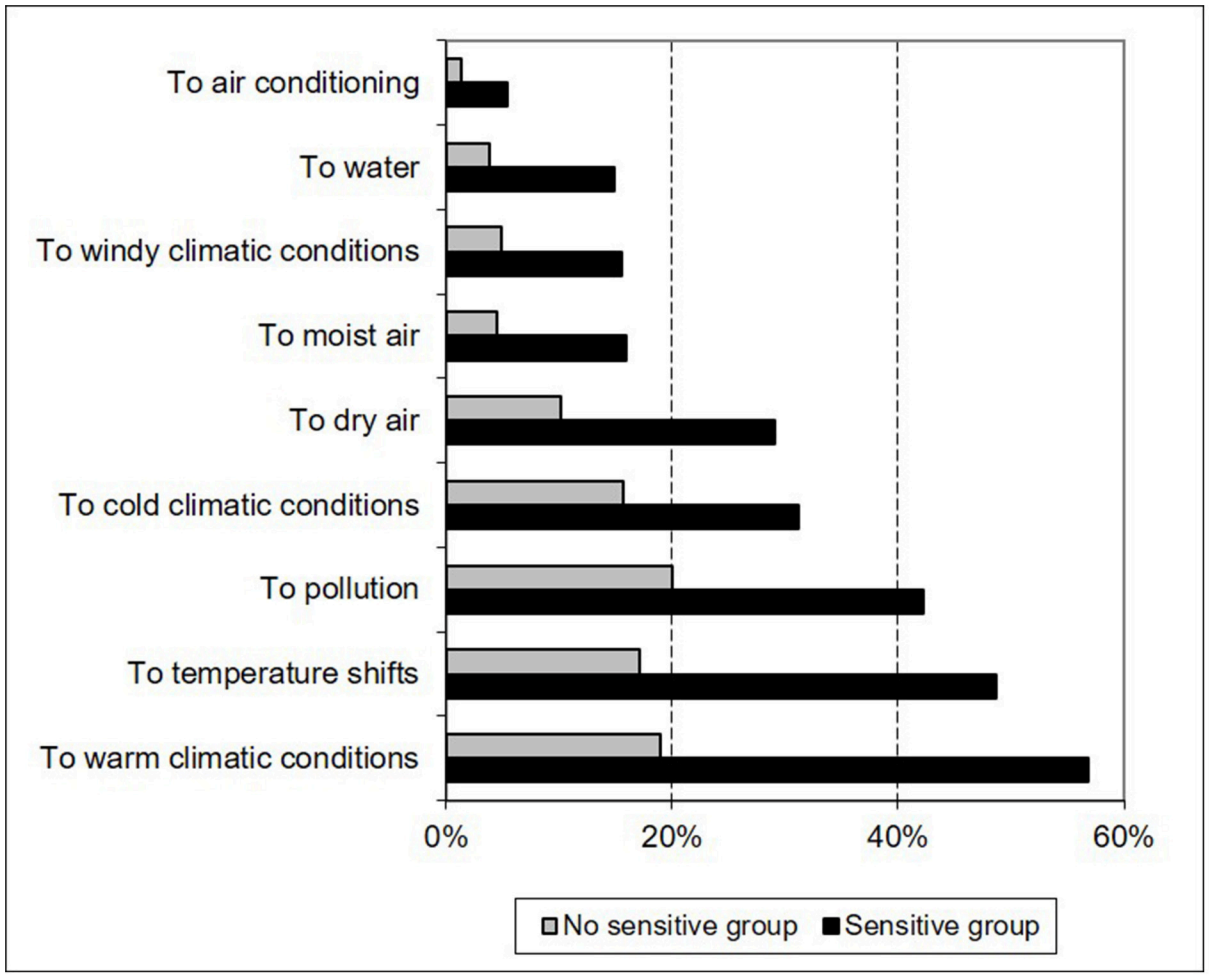
Climatic or environmental factors of sensitive skin reported in patients with sensitive skin.

In a series of questions regarding the onset of a rash, tingling, or irritation in the presence of various factors, such as emotional issues, cold, heat, sun, cosmetics, dry air, air-conditioning, water, pollution, and temperature variations, respondents with “sensitive” or “very sensitive” skin responded “yes” more often than others did: in particular, three to two times more often for temperature shifts (48.7 vs. 17.1%), water (14.95 vs. 3.7%), windy climatic conditions (15.5 vs. 4.9%), pollution (49.2 vs. 20%), air-conditioning (5.4 vs. 1.3%), dry air (29.1 vs. 10.1%), and cold weather (31.2 vs. 15.6%) (Figure 4).

### Skin Sensitivity Related to Cosmetics and Food Intake

Two questions assessed sensitivity to cosmetics and food intake. Respondents with “sensitive” or “very sensitive” skin reported skin sensitivity to cosmetics that was 2 times as high (14.7 vs. 5.5%, *p* < 0.001) as that of respondents who reported a greater skin sensitivity associated with food (spices) (19.6 vs. 6.2%, *p* < 0.0001).

### History of Consultation With a Dermatologist

The people in the “sensitive skin” group had previously seen a dermatologist more frequently than those in the “non-sensitive skin” group had (15 vs. 2.9%, *p* < 0.0001).

## Discussion

This study is the first to focus on sensitive skin among Indian people and is of particular interest for two reasons: (i) it was conducted on a representative sample of the Indian population by a recognized polling institute, and (ii) the methodology used was identical to that used in sensitive skin assessment studies conducted in Europe, the US, Brazil and Russia, making it possible to draw certain comparisons.

In the present study, approximately 27.9% of men and 36.7% of women reported having “sensitive” or “very sensitive” skin in response to the first question. In comparison, approximately 52.8% of Japanese men and 56% of Japanese women answered “sensitive” or “very sensitive” in response to this same question (12). In total, 32.4% of the sample of the Indian population (of both sexes) reported having “sensitive” or “very sensitive” skin, representing slightly less than one Indian person in 3 and a significant proportion of the Indian population.

The prevalence of sensitive or very sensitive skin among Indians is very similar to that in Spain, Greece, Portugal, and Brazil but lower than that in other countries (Japan, the US, France, and Italy). Because we used the same methodology to evaluate the frequency of skin sensitivity as in our previous studies (8–13), we could conduct a comparison of the prevalence of skin sensitivity in different countries (Table 1), which showed many differences. It is difficult to determine whether these differences are due to cultural, linguistic, genetic, environmental, or climatic factors.

**Table 1:** Comparison of frequencies of sensitive skin in different countries [data from this study and from (14)].

	**Sensitive (%)**
Japan	54.5
Italy	53.0
France	51.9
USA	44.6
Russia	39.7
Germany	35.6
Brazil	34.2
Spain	31.6
Switzerland	30.8
India	32.4
Greece	29.8
Portugal	27.4
Belgium	25.8

Geographical differences within the same country were also found in previous studies (8–13). To the hypotheses that we suggest regarding differences among countries, we could add that different regions within India were defined arbitrarily in the study, which could be a major bias.

To the questions regarding the onset of a rash, tingling, or irritation in the presence of various factors, such as emotional issues, cold, heat, sun, dry air, air-conditioning, water, pollution, and temperature variations. Indian respondents with “sensitive” skin responded “yes” two to three times more often than respondents in the “non-sensitive skin” group did. All of these data confirm that “skin sensitivity” was understood as defined by the medical community (1–3, 5, 15).

A limitation of this study is that sensitive skin was not diagnosed by doctors, but rather declared by subjects. However, sensitive skin is a condition that is diagnosed through subjective symptoms reported by subjects (3) and surveys (16) are therefore a suitable method for conducting an epidemiological study on sensitive skin. Moreover, all epidemiological studies on sensitive skins used this method (7–13, 17, 18). The use of a representative sample of the population is useful for giving extrapolated results (16) although statistics could be eventually overestimated or underestimated (14).

In summary, this is the first study of sensitive skin among the Indian people. This study was conducted on a representative sample of the population by a recognized polling institute. As India counts one billion of persons, and the mean frequency of sensitive skin is 32.3%, we can extrapolate that ~400 millions of Indians appear to have sensitive or very sensitive skin. This population is probably much larger than that spontaneously complaining of the condition to dermatologists. Dermatologists should therefore routinely question their patients about sensitive skin.

## Author Contributions

CT and LM contributed conception and design of the study. CT performed the statistical analysis. EB and CT wrote the first draft of the manuscript. All authors contributed to manuscript revision, read, and approved the submitted version.

### Conflict of Interest Statement

CT is employee of the Pierre Fabre Group. LM has been or is currently a consultant for Bioderma, Clarins, Expanscience, Johnson & Johnson, L'Oréal, Nestlé Skin Health, Pierre Fabre, Solabia, and Uriage. EB was speaker for Bioderma.
